# Impact of lipid binding on the tertiary structure and allergenic potential of Jug r 3, the non-specific lipid transfer protein from walnut

**DOI:** 10.1038/s41598-019-38563-1

**Published:** 2019-02-14

**Authors:** Pawel Dubiela, Rebecca Del Conte, Francesca Cantini, Tomasz Borowski, Roberta Aina, Christian Radauer, Merima Bublin, Karin Hoffmann-Sommergruber, Stefano Alessandri

**Affiliations:** 1 Department of Pathophysiology and Allergy Research, Medical University of Vienna, Vienna, Austria; 2 CERM & Department of Chemistry, University of Florence, Florence, Italy; 3 Jerzy Haber Institute of Catalysis and Surface Chemistry, Polish Academy of Sciences, Krakow, Poland; 4 Department of Statistics, Computer Science, Applications “G. Parenti” (DiSIA), Florence, Italy

## Abstract

Plant non-specific lipid transfer proteins type 1 (nsLTP1) are small basic proteins with a hydrophobic cavity able to host a number of different ligands: i.e. fatty acids, fatty acyl-CoA, phospholipids, glycolipids, and hydroxylated fatty acids. However, ligand binding specificity differs among nsLTPs. Within this protein family, Jug r 3 from walnut has been identified as a major allergen. So far, data on the structural characterization of Jug r 3 and its lipid binding capacity are lacking. We report the results from a fluorescence-based ligand-binding assay and ligand-based NMR experiments, to study the binding interactions between Jug r 3 and the 18-carbon monounsaturated oleic acid. Furthermore, protein-based NMR experiments were employed to detect the oleate binding site of Jug r 3. The NMR data were used to dock the oleate molecule into the structural model of Jug r 3. Finally, the impact of the interaction on the allergenic potential of Jug r 3 was investigated by IgE ELISA with 6 sera from walnut allergic patients. Our data corroborate the hypothesis of direct impact of food-derived matrix on the IgE reactivity of nsLTPs.

## Introduction

Allergy was found to be the most common chronic disease in Europe and according to recent studies, its prevalence is increasing^1,2^. Food allergy symptoms range from mild (oral allergy syndrome) to severe and potentially life-threatening reactions (anaphylaxis). The disease is due to an IgE-mediated adverse response to specific proteins called allergens. Upon first contact of genetically predisposed individuals with an allergen, sensitization takes place and allergen specific IgE antibodies are produced. Re-exposure to the allergen starts the eliciting phase characterized by the onset of allergic symptoms^2^. Unfortunately, no immunotherapy is currently available, thus the avoidance of the causative allergenic food source is the method of choice for the patient^2^.

Interestingly, allergens were found in only 2% of all sequence-based and 5% of all structural protein families suggesting common biochemical features such as lipid binding^3^. Non-specific lipid transfer proteins (nsLTPs) have been discovered in 1975 and received their name from the ability to transfer lipids in plants^4^. They are classified as pathogenesis-related proteins (PR-14) and in parallel belong to the prolamin superfamily^5^, nsLTPs are small and soluble, cysteine-rich proteins^6^. They possess four α-helices, which are stabilized by four conserved disulfide bridges formed by an eight-Cys motif with the general form C-Xn-C-Xn-CC-Xn-CXC-Xn-C-Xn-C. The disulfide bridges promote the folding of the nsLTP into a very compact structure, which is extremely stable to heat and denaturation agents^7^.

In the 1990’s clinicians also became interested in nsLTPs since they were identified as relevant plant food allergens^8^. Based on the International Union of Immunological Societies database (www.allergen.org) up to date 43 nsLTPs were identified as allergens. Among them, 37 are food allergens (as of 24.07.2018). Sensitization to nsLTPs is characterized by geographical differences, happens via different routes and is often associated with severe symptoms of food allergy^9–11^.

Studies on the cross-reactivity of nsLTPs showed that most Rosaceae-allergic and nsLTP mono-sensitized patients experience severe reactions also after ingestion of botanically unrelated plant-derived foods. The most frequently reported causes of allergic symptoms by cross-reactivity with Rosaceae were tree nuts (hazelnut, walnut)^12,13^.

nsLTPs are classified into two types named LTP1 and LTP2. These types differ by their molecular mass as LTP1s have about 90 amino acids and LTP2s have about 70 amino acids, respectively^14^. Up to date, the 3D structures of nsLTP1 from rice, barley, corn, wheat, peach, tobacco, hazelnut and mung bean have been solved by either X-ray crystallography or NMR^15–22^. The common feature of the structure is the presence of a cavity that can bind small hydrophobic molecules. This function is relevant for several physiological roles within the plant, such as the stabilization of membranes, cell wall organization, and signal transduction^7^. The tunnel can host different kind of ligands, i.e. fatty acids, fatty acyl-CoA, phospholipids, glycolipids, hydroxylated fatty acids and prostaglandin B2^6,23–27^. However, this binding capacity varies among different members of the nsLTP family, and depends on the specific characteristics of their tertiary structure. Notably, some nsLTPs can bind one or two lipid molecules simultaneously while others are unable to bind and transport free lipids or completely lack the internal lipid-binding cavity as was comprehensively reviewed by Liu *et al*.^7^.

Jug r 3 from walnut is an nsLTP1 identified by Pastorello *et al*. as the major allergen in an Italian cohort of walnut allergic patients. From a total of 46 patients with mild or severe clinical symptoms, 36 (78%) individuals revealed IgE reactivity to Jug r 3^28^.

So far, the Jug r 3 structure has remained unresolved and there are no reports regarding its lipid binding capacity. Interestingly, it exhibited IgE cross-reactivity with the major allergen from peach, Pru p 3, suggesting that both proteins share relevant IgE-binding epitopes^28^.

It has been shown that the interaction between free fatty acids and nsLTPs affects the local conformation of the protein^29^. We have recently demonstrated that in Pru p 3 such interactions increase also the allergenic potential of the protein^30^. However, experimental structural data on the atomic level remained limited.

Therefore, we performed an NMR interaction study and data driven docking calculations in order to characterize the complexes between Jug r 3 and selected free fatty acids and to obtain a structural model of the major walnut allergen Jug r 3 in complex with oleate (OLE). Furthermore, we tested the impact of structural changes induced upon ligand binding on the IgE binding activity of the allergen. The structural characterization of the Jug r 3-OLE complex here reported is relevant, not only for investigating the function of the protein, but potentially also for shedding further light on the role of lipids in allergy. Moreover, the comparison of this structure with other members of the nsLTP family may help in elucidating the importance of these proteins regarding their allergenic activity.

## Results

### Characterization of Jug r 3

Recombinant Jug r 3 was produced in the *Pichia pastoris* expression system as secreted protein with a yield of 5 mg/l and purified by using standard chromatography techniques. SDS-PAGE showed that the protein was highly pure and had a molecular mass of ~13 kDa (Supplementary Figure S1A). MALDI-TOF MS analysis (Fig. S1B) provided a mass of 9,646.9 Da corresponding to the theoretical mass of Jug r 3 (9,185.7 Da) with four additional N-terminal residues (EAEF) derived from the signal sequence cleavage site. CD spectroscopy revealed a spectrum compatible with the characteristic α-helical structure of nsLTPs^22,31^, with two negative extremes at 209 nm and 221 nm (Fig. S1C).

### Fluorescent ligand displacement assay (ANS assay)

**Figure 1:**
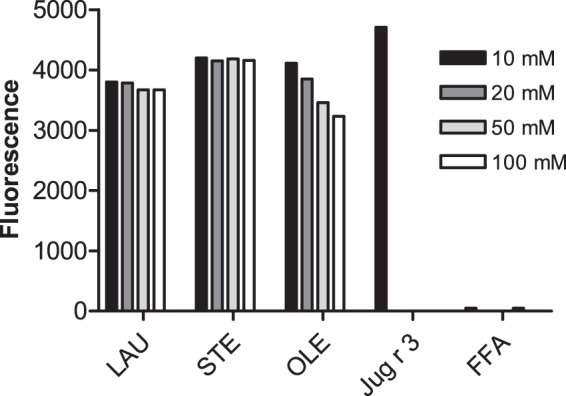
ANS displacement assay. Concentration dependent reduction of ANS binding to Jug r 3 pre-incubated with free fatty acids (FFA): oleate (OLE); stearate (STE); laurate (LAU). Positive control: Jug r 3; Negative control: FFA.

Pre-incubation of Jug r 3 with individual unsaturated and saturated free fatty acids revealed different binding capacity of the protein to oleate (OLE), stearate (STE) and laurate (LAU). A significant dose-dependent reduction of the signal was observed for OLE but not for other tested ligands. Pre-incubation of OLE and Jug r 3 revealed a reduction of the fluorescent signal of 13%, 18%, 27% and 31% at a protein:ligand ratio of 1:1, 1:2, 1:5 and 1:10, respectively (Fig. 1).

### NMR-based ligand binding assay

**Figure 2:**
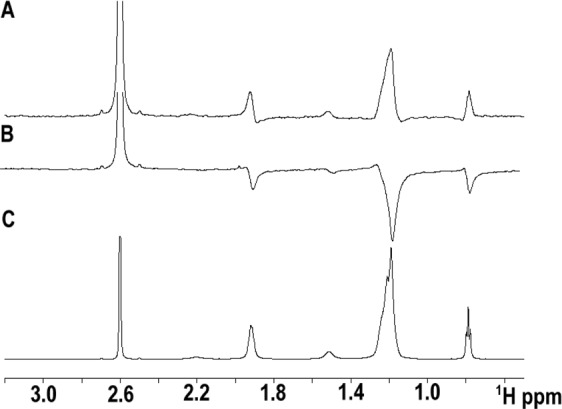
(**A**) 1-D WaterLOGSY spectrum of 80 µM OLE alone; (**B)** 1-D WaterLOGSY spectrum of OLE in the presence of Jug r 3 (8 µM); (**C**) ^1^H spectrum of OLE (80 µM). The signal at 2.57 ppm is caused by DMSO.

To corroborate the results of the ANS assay, we performed W-LOGSY (water-ligand observed via gradient spectroscopy) NMR experiments, a frequently applied 1D NMR technique for the detection of protein-ligand interactions. W-LOGSY spectra experiments were acquired for OLE and STE in the presence or absence of Jug r 3 as they showed the highest and the lowest binding capacity, respectively (Fig. 2). Comparison of these two spectra indicates that Jug r 3 was able to bind OLE as shown by an inversion of the signal. In contrast, no significant interaction of Jug r 3 with STE was detected (Fig. S2). NMR experiments therefore confirmed the binding specificity observed in the ANS assay.

### Jug r 3-oleate NMR interaction experiments

The ^1^H-^15^N HSQC spectrum of OLE-free Jug r 3 shows well dispersed resonances indicative of a folded protein. Eighty of the expected eighty-nine ^15^N backbone amide resonances were assigned (BMRB entry 27637). The amide resonances are missing for residues 4–7 and 81–83.

**Figure 3:**
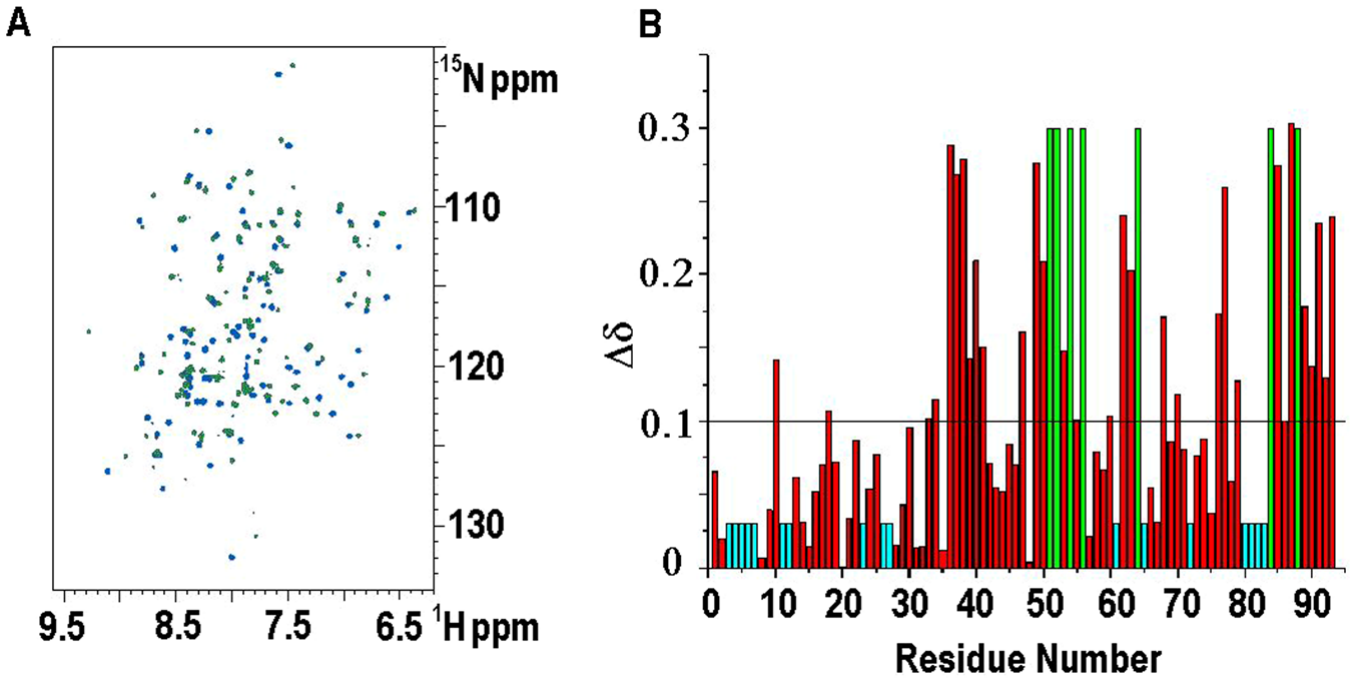
(**A**) Superimposition of the [^1^H, ^15^N] HSQC spectra of apo-Jug r 3 (blue) and OLE-bound Jug r 3 (green). (**B**) Bar graphs of the combined chemical shift perturbation of amide NH signals of apo-Jug r 3 compared with OLE-bound Jug r 3(ΔδHN) as a function of sequence. Residues for which we were unable to assign the NH signals in the free protein are coloured in cyan, residues that showed chemical shift perturbations upon addition of OLE but for which we were unable to assign NH NMR signals in the bound form are colored in green (residues E51, C52, K54, T56, N64, S84, T88). The mean value is shown as a continuous line (∆δHN ≥ 0.10 ppm).

**Figure 4:**
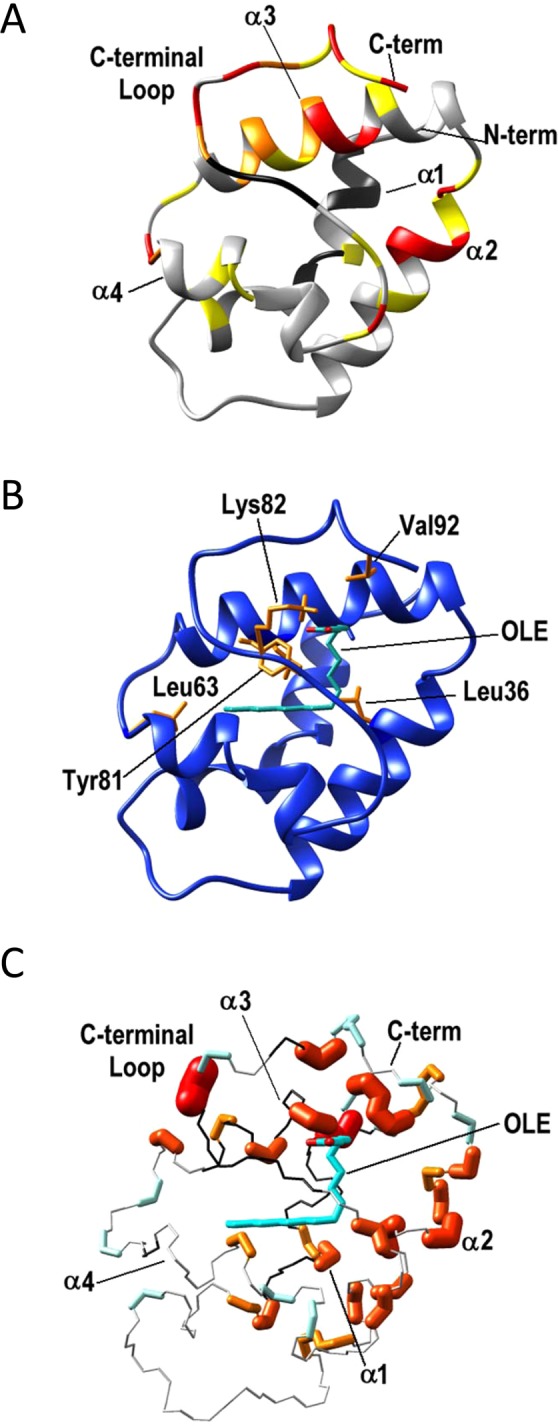
(**A**) Residues experiencing chemical shift perturbation as result of Jug r 3-OLE adduct formation are mapped onto the Jug r 3 model. Residues experiencing the most significant chemical shift variation (mean value + one standard deviation; ∆δHN ≥ 0.18 ppm) are shown in red, in yellow residues experiencing chemical shift variation lower than the latter but higher than the mean value (0.10 ppm ≤ ∆δHNav ≤ 0.18 ppm). Residues that experience chemical shift perturbation upon addition of OLE but for which we are not able to assign NH backbone NMR signals in the bound form are shown in orange and residues for which we were unable to assign the NH backbone NMR signals in the free form are shown in black. (**B**) Top ranking structure of the Jug r 3-OLE structural model obtained with HADDOCK program. Residues that establish contacts with OLE (cyan) in the majority of the conformers are shown in orange. (**C**) Residues which experience local mobility are mapped into the OLE-bound Jug r 3 structural model. Residues with R_2_ higher than the average value are shown in red/orange; the radius of the atom bonds is proportional to the magnitude of the R_2_ value. Residues with ^15^N{1H}-NOEs lower than the average are shown in sky blue. Residues for which we were unable to assign the NH backbone NMR signals in both, free and bound forms are shown in black.

The addition of increasing amounts of OLE to ^1^H^15^N labeled Jug r 3 protein up to a Jug r 3:OLE molar ratio of 1:6 induced chemical shift changes (Fig. 3A), which were monitored for the backbone amide groups through ^1^H-^15^N HSQC spectra. The OLE-bound and OLE-free forms of the protein exchanged with each other at a slow rate with respect to the resonance frequency differences in the two species (Fig. S3). The residues of Jug r 3 affected by OLE binding (Fig. 3B) were mapped onto the structural model of Jug r 3 (Fig. 4). The identified OLE binding site included the C-terminal region of helix α2, almost the complete helix α3, the loop between helices α3 and α4 (loop3) and the C-terminal loop region.

### Computer modelling of the Jug r 3-oleate complex

The I-TASSER server was used to generate a homology model of the unliganded Jug r 3 protein. The latter consists of an α-helical structure with four helices connected by short loops and stabilized by the presence of four disulphide bridges. This model was subsequently refined by energy minimization and molecular dynamics (MD) in the presence of the OLE molecule. The resulting Jug r 3 structure showed that the C-terminal loop region moved towards the solvent with respect to the model of the OLE-free Jug r 3 where the same region was close to the core of the molecule. The coordinates of the two molecules, i.e. Jug r 3 and OLE, were split and used as input, together with the NMR data (Table S2) to run docking calculation with HADDOCK2.2 program^32^. These calculations resulted in the model of Jug r 3-OLE complex shown in Fig. 4B, which represents the top-ranking cluster for energetic and scoring functions. This lowest energy cluster experienced also the higher number of conformers. The list of Jug r 3 residues contacting OLE in the best five structures of the selected cluster is given in Table 1. The obtained model shows that the hydrophobic tail of the ligand is embedded in the core of the Jug r 3 while the carboxylate portion is turned towards the solvent. The conformation of the hydrophobic tail of OLE in the protein cavity presents some variability but it establishes contacts with hydrophobic aliphatic residues Leu36 (helix α2), Leu53 (helix α3), Leu63 (loop3), Ala68 (helix α4) and Val92 (C-term loop) and with the aromatic residue Tyr81 (C-terminal loop) in the majority of the conformers. On the contrary, the negative charged carboxylic end points towards the exterior of the cavity and establishes contacts with Lys82 (C-terminal loop) in more than half of the conformers.

**Table 1:** Residues involved in the interaction with OLE as derived from the NMR analysis are presented in the column “NMR”.

Residues of Jug r 3	NMR	Cluster1 OLE
Ser 10	*	
Leu 18	a	*
Leu 36	*	*
Arg 46	a	*
Ala 49	*	
Cys 50	*	*
Leu 53	*	*
Leu 63	*	*
Ala 68	*	
Val 79	*	*
Pro 80	b	*
Tyr 81	NA	*
Val 92	*	*

Residues for which intermolecular non-bonded contacts with OLE are present in at least three of the best five model structures are reported in the column “Cluster1 OLE”. The occurrence of interaction in a cluster is indicated by an asterisk (*).

^a^These residues have not been reported due to spectral overlap of their backbone amides or as they have a chemical shift perturbation lower than 0.16 ppm. ^b^Proline residue has no amide proton. ^NA^Not assigned residues in free Jug r 3.

### IgE ELISA

**Figure 5:**
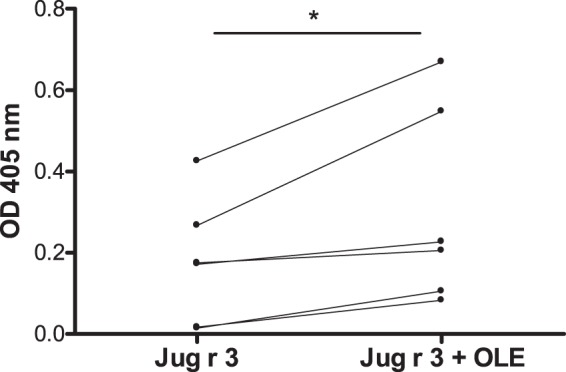
Jug r 3-OLE interaction and impact on IgE binding. Data from 6 patients’ sera tested in duplicates with recombinant Jug r 3 alone and with OLE. The P value refers to the comparison of the median between allergen alone and in the presence of ligand. *p ≤ 0.05 (paired two-tailed t-test).

To test whether the interaction of recombinant Jug r 3 with OLE influences its IgE binding capacity, we used sera from 6 patients sensitized to Jug r 3 (Table 2). When compared to the allergen alone, pre-incubation of recombinant Jug r 3 with OLE significantly (p = 0.03) increased the IgE binding capacity of all tested sera ranging from 1.2 to 7.5 fold increase (Fig. 5). The largest differences were observed with sera no. 1 and 3 while sera no. 4 and 6 showed the smallest increase of IgE binding capacity, respectively.

**Table 2:** Clinical data of 6 selected walnut allergic patients.

Patient no	Sex	Age	IgE specific to walnut (kU/L)	AE	URT	RHIN
1	F	19	4.8	+	+	
2	F	48	2.9	+	+	
3	F	23	6.4		+	
4	M	29	4.3		+	
5	M	21	3.8			+
6	M	24	9.2	+	+	

AE, angioedema; URT, urticaria; RHIN, rhinitis.

## Discussion

Non-specific lipid transfer proteins are important food allergens responsible for severe allergic reactions in predisposed individuals^9,10,33^. Although tertiary structures of some nsLTPs have already been resolved, there is still a lack of experimental knowledge regarding the impact of ligands on the protein 3D structure. Since it has been shown that the ligand binding specificity can vary among protein family members^10,20^ and that this is an important factor regarding nsLTP allergenicity^30^, we investigated the ligand binding ability of the major allergen from walnut, Jug r 3. We also investigated the interaction of Jug r 3 with OLE by NMR technique and an *in silico* docking program to model the Jug r 3-OLE complex.

**Figure 6:**
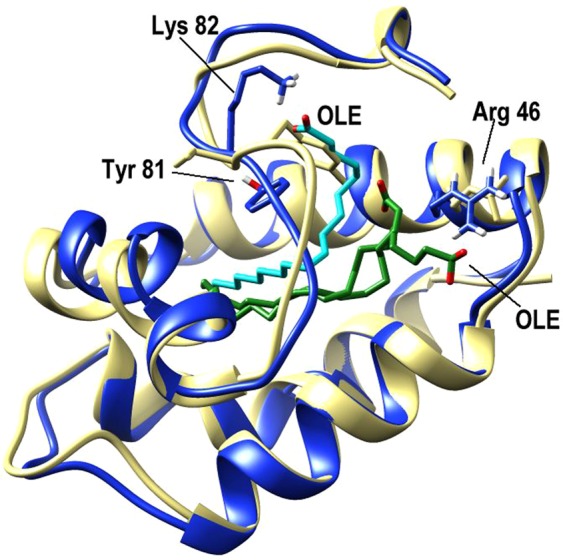
Superposition of the Jug r 3-OLE model (blue and cyan) with the X-ray structure of the homologue from maize, OLE-Zea m 14 (khaki and green), PDB 1fk5.

In agreement with the literature, the Jug r 3 model shared the typical fold of nsLTP1 comprising a four helices bundle with a long C-terminal segment and 4 conserved disulfide bridges. The Jug r 3–OLE complex showed a large internal hydrophobic cavity which is fully occupied by the OLE molecule. The OLE is positioned similarly to that of the complex with Zea m 14, an allergenic nsLTP1 from maize (*Zea mays*; PDB 1fk5)^17^. In both cases the hydrophobic tails point towards loop3 of the protein but their polar heads have different orientations (Fig. 6). In the X-ray structure of Zea m 14 the electron density suggested two different conformations for OLE binding. In the first one, the carboxylate group of OLE molecule points towards the C-terminal region similarly to the Jug r 3 complex and establishes a hydrogen bond with the OH group of Tyr81. In the second conformation, the charged head group of OLE is more exposed on the protein surface and forms hydrogen bonds with Asn37 (α2, maize numbering) and Arg46 (α3).

According to our data, only OLE is able to bind Jug r 3, whereas STE and LAU showed no or modest interaction. The OLE molecule guarantees a high level of hydrophobic interactions optimized by the long C18 chain of the molecule and by the specific conformation due to the presence of the double bond. These features are not present in the saturated fatty acids LAU and STE. Indeed, the short C12 chain of the former does not permit a significant number of hydrophobic interactions in the protein cavity. The saturated C18 fatty acid STE has the optimal length for establishing such interactions, but the absence of the double bond does not stabilize the ideal conformation to form a significant number of contacts with the protein. These binding preferences are in line with the results for Pru p 3, the nsLTP1 from peach, which also preferentially binds longer-chain and unsaturated fatty acids than shorter and saturated fatty acids^29^. These data are also consistent with a previous observation that moss GPI-anchored nsLTPs prefer binding to unsaturated C18 fatty acids^34^. The plasticity of the hydrophobic cavity of nsLTP1 described in literature is another important factor for the protein-lipid interaction recognition^18,29^.

The ^15^N heteronuclear relaxation measurements performed on the OLE-bound Jug r 3, revealed that some residues located in the internal cavity, formed by the C-terminal loop, helix α2 and helix α3 are affected by local internal motions occurring on a fast timescale with respect to the overall re-orientational correlation time (τ_c_) of the molecule (Fig. S4 and residues shown in sky blue in Fig. 4C). The analysis of ^15^N relaxation measurements revealed also that many residues located in these regions had transversal R_2_ values higher than the average value, suggesting the presence of conformational exchange processes on the ms-μs timescale. In agreement, backbone NH of some residues located in helix α3 (E51, C52, K54 and T56) and in the C-terminal loop (S84 and T88) are even not detectable, likely as a consequence of an increased local mobility or solvent exchange (Figs 4C and 3B).

The existence of such flexibility in the region forming the internal cavity might play a role in the selection of the optimal conformation that facilitates the right lipid-protein contacts. Moreover, analysis of the published three-dimensional structures of nsLTP1s, revealed that the volume of the binding cavity depends on the presence of the bound lipid in agreement with the fact that the internal cavity does not retain a rigid structure but experiences conformational variability able to accommodate the lipids^15–22^. Our structural models (unbound Jug r 3 and Jug r 3-OLE complex), are in agreement with what has been reported in the literature as the C-terminal loop region changed its conformation upon lipid binding.

We performed *in vitro* studies on the IgE binding of Jug r 3. Our data showed an increased IgE binding capacity of Jug r 3-OLE complex in comparison to Jug r 3 alone. From these results we can propose that local mobility could lead to the exposure of additional IgE epitopes and thus directly affect the IgE binding activity of this protein as reported for Pru p 3^30^.

The structural characterization of the Jug r 3-OLE complex may have important implications to the exploration of innovative therapies which are based on the rational design of safe candidates for food allergy immunotherapy^35^. By targeting residues involved in ligand binding it may be possible to reduce the number of IgE epitopes and mitigate allergen cross-linking on mast cells and basophils. This innovative approach is only possible if detailed structural information of the allergen is available. In this context the NMR based characterization of the major walnut allergen here reported, suggests also a good agreement with the hypothesis that the food matrix plays an important role in food allergy by affecting the 3D structure of allergens.

## Material and Methods

### Protein production

Recombinant Jug r 3 was produced in the yeast *Pichia pastoris*. The protein sequence of mature Jug r 3 was retrieved from Genbank (Acc. no. EU780670.1), and optimization for *P. pastoris* codon usage as well as prediction of glycosylation sites was performed. The plasmid construct pPICZαA-Jug r 3 (ThermoFisher Scientific, Waltham, MA, USA) was linearized with SacI (New England Biolabs, Ipswich, MS, USA) and used to transform GS115 *P. pastoris* cells (ThermoFisher Scientific) by electroporation. Transformed cells were grown on yeast extract peptone dextrose (YPD) medium plates containing 100 μg/ml zeocin (Invivogen, San Diego, CA, USA) at 28 °C for 5 days. Multi copy screening was performed by replica plating of the positive cells on YPD with increasing zeocin concentration (up to 2,000 μg/ml). Positive transformants expressing Jug r 3 with the highest yield were cultivated in 200 ml minimal glycerol medium containing histidine for 1 day at 28 °C with shaking until the culture reached an OD_600_ of 2–3. Cells were harvested and transferred to Minimal Methanol + Histidine medium (1.43% yeast nitrogen containing base, 4 × 10^−5^% biotin, 1% methanol and 0.0004% Histidin) or Silantes Yeast-OD2 C and Silantes Yeast-OD2 CN medium (Silantes, Munich, Germany; for more information see Supplementary Information) containing 1% methanol or ^13^C-methanol and ^15^N, respectively. Cultivation was performed at 24 °C with shaking at 140 rpm to induce expression of non-labelled or single/double labelled protein, respectively. Methanol (at a final concentration of 1%) was added to the culture every 12 hours. After 5 days, the culture supernatant was collected by centrifugation (6,000 g for 20 min at 4 °C) and stored at −20 °C.

For subsequent purification, 200 ml of supernatant were dialyzed against binding buffer (20 mM sodium acetate, pH 6.5). After filtration through a 0.22 μm filter, the protein solution was applied to a 1 ml Mono S column (GE Healthcare, Little Chalfont, United Kingdom). Bound proteins were eluted by a linear gradient of NaCl (0–0.5 M) at a flow rate of 1 ml/min. Samples containing the protein of interest were applied on a RP-HPLC Jupiter C5 analytical column (Phenomenex, Torrance, CA, USA). The column was equilibrated with 10% acetonitrile, 90% water, 0.1% TFA and eluted by increasing the concentration of acetonitrile (up to 50%) with a flow rate of 1 ml/min. nsLTP containing fractions were analyzed by 15% SDS-PAGE, and the concentration of purified Jug r 3 was determined using the bicinchoninic acid assay according to the manufacturer’s protocol (ThermoFisher Scientific). Samples were stored at −20 °C.

### Protein characterization

The N-terminal sequence of recombinant Jug r 3 was determined using an Applied Biosystems Procise 491 sequencer (Applied Biosystems, Foster City, CA, USA). Purified protein (100 pmol) was adsorbed onto a Prosorb cartridge and subjected to sequence analysis. For mass determination, Jug r 3 was measured in linear mode on a MALDI-TOF mass spectrometer (Microflex, Bruker Daltonics, Bremen, Germany) using α-cyano-4-hydroxycinnamic acid as matrix.

Secondary structure analysis of purified Jug r 3 was performed by CD (circular dichroism)-spectroscopy on a Jasco J-810 spectropolarimeter (Jasco International Co., Hachioji, Tokyo) measured from 190 to 260 nm at 25 °C in 10 mM sodium phosphate, pH 7.5 in a 2 mm path length quartz cell. Spectra represent the average of four accumulations. The spectrum is represented as molar CD (with respect to moles of peptide bonds).

### Ligand binding

#### ANS displacement assay

The probe 1-anilinonaphthalene-8-sulfonic acid (ANS) is non-fluorescent in water but fluorescent in a hydrophobic environment with maximum emission at 456 nm. Recombinant Jug r 3 (10 μM) was incubated overnight at 4 °C with 3 different ligands: oleate (OLE; C18:1, where one indicates the presence of a double bond), stearate (STE; C18:0) and laurate (LAU; C12:0) at molar protein:ligand ratios of 1:1, 1:2, 1:5, and 1:10. Binding of ligands was monitored by adding 10 µM ANS and measuring the decrease of fluorescence relative to the protein sample without ligands. All samples were analyzed in triplicates. Purified Jug r 3 with ANS and ligands with ANS served as controls.

#### W-LOGSY (Water-Ligand Observed via Gradient Spectroscopy)

W-LOGSY NMR experiments were carried out using an Avance 700 Bruker spectrometer equipped with a cryo probe, operating at a proton resonance frequency of 700 MHz (11.7 Tesla) at 298 K. W-LOGSY is based on a transient nuclear Overhauser effect (NOE) experiment and implies transfer of magnetization via an intermolecular NOE and spin diffusion. Non-binders and binders are easily discriminated from each other because they give W-LOGSY signals of opposite sign. W-LOGSY NMR experiments were acquired for OLE and STE dissolved in H_2_O with 10% (v/v) hexadeuterodimethyl sulfoxide. Recombinant Jug r 3 stock solution was prepared in 20 mM sodium acetate buffer pH 6.5 containing 0.1 M NaCl with 10% (v/v) D_2_O. The allergen was mixed with the ligands at a molar ratio of 1:20 at a final protein concentration of 5 μM. The experiments were performed with a 180° inversion pulse applied over the water signal at ~4.7 ppm by means of a Gaussian-shaped selective pulse of 10 ms. Each W-LOGSY spectrum was acquired with 512 scans.

### Jug r 3 structure characterization

#### Jug r 3-oleate NMR titration

For the NMR titration a stock solution of OLE (2.5 mM) was prepared in 20 mM sodium acetate, 0.1 M NaCl, 10% DMSO (dimethyl sulfoxide), pH 5. This pH guaranteed the absence of aggregated molecules of OLE.

A Jug r 3 sample (in 20 mM sodium acetate, 0.1 M NaCl, pH 5) was titrated with increasing amounts of OLE up to a 1:6 Jug r 3:OLE molar ratio. The obtained NMR spectra were compared with those acquired on apo Jug r 3 sample containing 10% of DMSO. The combined chemical shift perturbation (Δ_HN_) of Jug r 3 NH backbone signals (Fig. 3) is given by the equation Δ_HN_ = {((H_Nfree_−H_Nbound_)^2^ + ((N_free_−N_bound_)/5)^2^)/2}^½^ ^36^.

#### NMR experiments for Jug r 3 backbone assignment

NMR experiments for the assignment of backbone resonances of OLE-free Jug r 3 were acquired on two apo ^13^C ^15^N labelled samples (0.2 mM) dissolved in 20 mM sodium acetate, 0.1 M NaCl at pH 6.5 and pH 5.0, respectively. NMR experiments on OLE-bound Jug r 3 were obtained from a ^13^C ^15^N labelled sample at pH 5. All NMR samples contained 10% (v/v) D_2_O (Sigma-Aldrich (Saint Louis, MS, USA).

The NMR experiments were performed on a 900 MHz Bruker spectrometer equipped with a triple resonance cryoprobe at 298 K. Details of the NMR experiments are reported in Table S1 of supplementary material. The backbone assignment of both, OLE-free and OLE-bound Jug r 3 were deposited on BMRB (entries 27637 and 27638).

#### Heteronuclear relaxation data

The dynamic properties of OLE-bound Jug r 3 were experimentally characterized through ^15^N relaxation measurements. ^15^N longitudinal R_1_ and transverse R_2_ relaxation rates and ^15^N{1 H}-NOEs were recorded at 298 K at 700 MHz, using a ^15^N,^13^C labelled sample (0.2 mM).

The average backbone ^15^N longitudinal R_1_ and transversal R_2_ relaxation rates and ^15^N{1H}-NOEs^37^ values were 1.8 ± 0.1 s^−1^, 9.0 ± 0.9 s^−1^ and 0.75 ± 0.09, (mean value ± one standard deviation) respectively (Fig. S4).

The correlation time for the molecule tumbling (τ_c_), as estimated from the R_2_/R_1_ ratio, was 5.50 ± 0.46 ns, consistent with the molecular weight of the monomeric protein and in agreement with the value of 5.18 ns predicted by the HYDRONMR program^38^. In this analysis, care was taken to remove from the input relaxation data those NHs having an exchange contribution to the R_2_ value or exhibiting large-amplitude internal motions on a time scale longer than a few hundred picoseconds as inclusion of these data would bias the calculated τ_c_ value^39^.

#### Modelling of Jug r 3

A model of the Jug r 3 structure was obtained using the fully automated protein structure homology modelling server I-TASSER. This server requires only the amino acid sequence as input data to generate a 3D model. Template selection, alignment, and model building are automatically performed by the server^40^. The obtained model was characterized by a scoring function C-score = 1.28, where the C-score is a confidence score for estimating the quality of predicted models by I-TASSER. C-score is typically in the range of [-5, 2]. Higher C-score value signifies a model with a high confidence and vice versa. The obtained value therefore reveals high accuracy of the I-TASSER result.

#### Molecular dynamic of the Jug r 3-Oleate complex

The homology model of Jug r 3 was used to define the initial coordinates of the protein for its molecular dynamic in the presence of OLE. The charged form of OLE was used in the calculation as previously reported for other fatty acids in^41^. The initial structure of the Jug r 3-OLE complex was prepared by first manually docking OLE into the hydrophobic cavity and then solvating the complex in a periodic box extending 10 Å from the complex surface in each direction filled with explicit water molecules described with the TIP3P model. MD simulations were conducted with the Amber12 suite^42^ with a GPU versions of the PMEMD program^43^. The protocol used has been extensively described in Supplementary Methods.

#### Docking calculations

Docking calculations were performed with HADDOCK2.2 (ttp://www.bonvinlab.org/education/HADDOCK-binding-sites/). Two sets of restraints obtained from the experimental NMR data were created and used at different stages of the docking calculation. In particular, in the rigid-body docking, residues of Jug r 3 that showed a meaningful chemical shift perturbation upon complex formation (Table S2) were defined as active, together with OLE. This ensures that the ligand is properly drawn inside the binding site. For the subsequent flexible refinement stages, only the binding region of Jug r 3 was defined as passive and OLE as active. This ensured that the ligand was allowed to fully explore the binding site of the protein^43^. Semi-flexible regions of the protein were defined, based on the active residues plus two preceding and following residues each. Moreover, the stretch 76–93 was defined as fully flexible during the entire docking protocol except for the rigid body minimization. All the parameters used to run HADDOCK calculations are reported in Supplementary Methods.

The top-ranking HADDOCK2.2 clusters (based on HADDOCK score) were manually analyzed and subjected to a per-cluster re-analysis following the protocol reported in http://www.bonvinlab.org/software/haddock2.2/analysis/#reanal. Seven clusters were obtained and ranked according to their HADDOCK score. From the analysis of the energetic and scoring functions, it resulted that only one is meaningful as it shows the lowest HADDOCK score value and better fulfills experimental data. Energy parameters (van der Waals energy, electrostatic energy, desolvation energy, and the penalty energy due to violation of restraints) for the top ranking cluster are reported in Supplementary Table S3. The structural models belonging to this cluster were visualized using UCSF CHIMERA^43^.

### Assessment of IgE binding activity of Jug r 3

#### Patients

Serum samples were obtained from 6 walnut allergic patients (3 females and 3 males with a mean age of 27.3; Table 2) and 4 non-atopic donors. All of the patients were recruited based on previously experienced food allergic symptoms (angioedema, urticaria, rhinitis) after walnut consumption and classified by allergologists as clinically allergic to walnut. Standardized interviews were conducted to assess allergic symptoms to walnut. Subjects were tested by ImmunoCAP and/or skin prick test to walnut extract. The study was approved by the Ethic’s Committee of the Medical University of Vienna (EK1534/2017) and conducted in accordance with the Declaration of Helsinki. Patients gave written informed consent. More detailed information about patients’ sera is shown in Table 2. 

#### IgE ELISA

To ensure the binding of the protein in the native state to the ELISA (Enzyme-linked immunosorbent assay) plate, Thermo Scientific™ Nunc™ Immobilizer™ Amino surface plates were used. Plates were incubated with 2 μg of purified recombinant Jug r 3 (2 μM) in 100 mM sodium carbonate, pH 9.6 at room temperature (RT) for 1 hour. Remaining electrophilic groups were quenched by reaction with 10 mM ethanolamine. Sixty μM of OLE were added and incubated overnight at 4 °C. According to the manufacturer’s protocol subsequent steps were performed. After washing, patients’ serum samples (1:10 diluted in TBST/0.5 BSA) were added to the wells and incubated ON at 4 °C. As negative controls, normal human serum (NHS), and OLE were tested in parallel. Afterwards AP-conjugated mouse anti-human IgE antibody (1:1000; 2 h RT; BD Biosciences) was added. Bound IgE was detected by colorimetry using SIGMA FAST^TM^ p-nitrophenyl phosphate substrate tablets (Sigma-Aldrich) and measured at 405 nm. The mean value of the negative controls plus 2x standard deviations were subtracted. The two tailed paired t-test was used for comparison of IgE binding to Jug r 3 with Jug r 3-OLE, respectively. P-values below 0.05 were considered statistically significant. Analyses were performed with GraphPad Prism (GraphPad Software, La Jolla, CA, USA). All sera were tested in duplicates.

## Supplementary information

Supplementary information

## Data Availability

The datasets generated during the current study are available from the corresponding author upon request.
