# Supplementary material for: Impact of lipid binding on the tertiary structure and allergenic potential of Jug r 3, the non-specific lipid transfer protein from walnut
Source: Sci Rep. 2019 Feb 14;9:2007. doi: 10.1038/s41598-019-38563-1 (PMC6376136; doi:10.1038/s41598-019-38563-1)
Supplement: Supplementary file 1 — Supplementary information [file 41598_2019_38563_MOESM1_ESM.docx]

**Supplementary information**

**Impact of lipid binding on the tertiary structure and allergenic potential of Jug r 3, the non-specific lipid transfer protein from walnut**

Pawel Dubiela, PhD^1§^ , Rebecca Del Conte, PhD^2§^ , Francesca Cantini, PhD^2§^, Tomasz Borowski, PhD^3^, Roberta Aina, PhD^1^, Christian Radauer, PhD^1^, Merima Bublin, PhD^1^, Karin Hoffmann-Sommergruber, PhD^1*^ and Stefano Alessandri, PhD^4^

^1^Department of Pathophysiology and Allergy Research, Medical University of Vienna, Vienna, Austria

^2^CERM & Department of Chemistry, University of Florence, Florence, Italy

^3^Jerzy Haber Institute of Catalysis and Surface Chemistry, Polish Academy of Sciences, Krakow, Poland

^4^Department of Statistics, Computer Science, Applications "G. Parenti" (DiSIA), Florence, Italy

^*^Corresponding author: Karin Hoffmann-Sommergruber PhD, Assoc. Prof.

^§^These authors contributed equally to this work

E-mail: karin.hoffmann@muv.ac.at

Department of Pathophysiology and Allergy Research

Medical University of Vienna

AKH-EBO 3Q

Waehringer Guertel 18-20

A-1090 Vienna, Austria

fax:+43-1-40400/51300; tel:+43-1-40400/51040

**SUPPLEMENTARY METHODS**

**Protein production**

Double labelled recombinant Jug r 3 for further structural analyses was produced in *Pichia pastoris* using the Silantes Yeast-OD2 CN labelled medium (Silantes, Munich, Germany, product nr: 111601402; ^13^C, ^15^N (98%)).

**Molecular dynamic of the Jug r 3-Oleate complex**

Molecular force fields of OLE were calculated with the general AMBER force field GAFF. OLE was first optimized in a fully extended conformation in vacuum at the B3LYP/6-31G(d,p) level and then the electrostatic potential around it was computed at the level that is consistent with the GAFF FF, i.e. HF/6-31G(d). Quantum chemical computations were performed with the Gaussian 09 suite (http://gaussian.com/g09citation). From the electrostatic potential, atomic charges were fitted with the use of antechamber and RESP programs from the AmberTools package[^39^](#_ENREF_39)^,^[^40^](#_ENREF_40). Amber12 molecular dynamic simulations of the Jug r 3-OLE complex were performed following the here reported protocol: i) the system was initially minimized for 5000 steps fixing the position of the protein; ii) the system was minimized for a further 5000 steps with a 10 kcal/mol Å^2^ harmonic restraint on the protein and then 10,000 steps of an unrestrained minimization; iii) the minimized system was subsequently heated up from 0 to 300 K during a 50 ps NV (conical ensemble) dynamics, and then its density was equilibrated in a 0.5 ns NPT (isobaric–isothermal ensemble) dynamics; in both steps protein backbone atoms were restrained with 1 kcal/mol Å^2^. Subsequent unrestrained NPT (T = 300 K, p = 1 atm) dynamics simulation spanned 150 ns with a snapshot saved every 10 ps. Integration time step was 2 fs. The SHAKE algorithm was used to constrain bonds of hydrogen atoms. Temperature and pressure were controlled with the Langevin dynamics and the isotropic position scaling algorithm, respectively[^39^](#_ENREF_39).

**Docking calculations**

Docking calculations were performed with HADDOCK2.2 using the here reported parameters. One thousand structures of the complex were generated in the initial rigid body docking calculation phase, and the best two hundreds in terms of total intermolecular energy were further submitted to the semi-flexible simulated annealing and final refinement in water. The initial temperatures for the second Torsion Angle Dynamics (TAD) cooling step with flexible side chain at the interface and for the third TAD cooling step with fully flexible interface were set to 500 K and 300 K, respectively. The constant of the intermolecular interactions for rigid body docking was scaled down to 0.01. Moreover, the number of molecular dynamics steps for rigid body high temperature TAD and for the first rigid body cooling stage were both set to 0 to favor the interaction with OLE. Finally, the weight for the van der Waals it0 scoring (w_vdw_0) was changed to 0.

**SUPPLEMENTARY TABLES**

**Table S1: Acquisition parameters for NMR experiments performed on OLE-free and OLE-bound Jug r 3.**

| **Experiments^a^** | **Dimension of acquired data** | | | **Spectral width**  **(ppm)** | | | **N^b^** |
| --- | --- | --- | --- | --- | --- | --- | --- |
|  | **t_1_ t_2_ t_3_** | | | **F_1_ F_2_ F_3_ F_3_** | | |  |
| ^1^H-^15^N-HSQC^e^ | 256(^15^N) | 2048(^1^H) |  | 36 | 16 |  | 2 |
| ^1^H-^13^C-HSQC | 165(^13^C) | 2048(^1^H) |  | 165 | 12 |  | 4 |
| CBCA(CO)NH^c,e^ | 96(^13^C) | 48(^15^N) | 2048(^1^H) | 80 | 36 | 12 | 32 |
| CBCANH | 96(^13^C) | 48(^15^N) | 2048(^1^H) | 80 | 36 | 12 | 32 |
| HNCA^c,e^ | 96(^13^C) | 48(^15^N) | 2048(^1^H) | 30 | 36 | 12 | 16 |
| HNCO | 96 (^13^C) | 48(^15^N) | 2048(^1^H) | 12 | 36 | 12 | 16 |
| HN(CA)CO | 96 (^13^C) | 48(^15^N) | 2048(^1^H) | 12 | 36 | 12 | 64 |
| ^15^N-edited [^1^H-^1^H]-NOESY^d^ | 128(^1^H) | 48(^15^N) | 2048(^1^H) | 12 | 36 | 12 | 16 |

^a^ All the triple resonance (TCI 5-mm) probes used were equipped with Pulsed Field Gradients along the z-axis. ^b^ number of acquired scans ^c^ These experiments were acquired on Jug r 3 samples at both, pH 5 and 6.5.  ^d 15^N-edited 3D NOESY-HSQC experiments were acquired with a mixing time value of 120 ms. All 3D and 2D spectra were processed using the standard Bruker software TOPSPIN 2.1 and analyzed through CARA (cara.nmr.ch).  ^e^ These experiments were acquired also on OLE-bound Jug r 3 sample at pH 5 in sodium acetate 20 mM and 0.1 M NaCl.

**Table S2: Amino acids of Jug r 3 whose amide signals experience chemical shift changes upon the addition of OLE.**

| S10, L36-A39, A41, Q47, A49-K54, T56, G62-N64, A68, G76, V77, V79, S84, T85, T87-K93 |
| --- |

**Table S3: Docking calculations.** Statistical analyses on the structural models of Jug r 3-OLE complex obtained through HADDOCK2.2 calculations. Averages (standard deviations are reported in parenthesis) were calculated over the best five model structures.

|  | **Cluster1 _OLE** |
| --- | --- |
| **HADDOCK Score ^a^** | -61.7 (1.9) |
| **RMSD (Å) ^b^** | 0.4 (0.2) |
| **Number of structures** | 92 |
| **BSA (Å^2^) ^c^** | 799 (14) |
| **Einter^e^** | -166 (5) |
| **Eelec^f^** | -145(5) |

^a^ HADDOCK score defined as a weighted sum of different energetic terms, such as: van der Waals energy, electrostatic energy, distance restraints energy, buried surface area, binding energy and desolvation energy. ^b^ Backbone RMSD from the lowest HADDOCK score structure in each cluster. Some individual energy terms are also reported: ^c^ Buried surface area, ^e^ binding energy, ^f^ electrostatic interaction energy.

**Supplementary Figure 1:** Production and characterization of Jug r 3. **A**. Coomassie stained 15% SDS-PAGE (Protein ladder and Jug r 3 derived from the same gel). **B**. MALDI-TOF MS analysis. **C**. Far-UV CD spectrum.

**Supplementary Figure 2:** W-LOGSY spectra of stearic acid (100 µM) either in absence (**A**) or presence (**B**) of Jug r 3 (10 µM). The 1D ^1^H spectrum of stearic acid (100 µM) is also reported (**C**). The NMR signal at 2.57 ppm is due to DMSO.


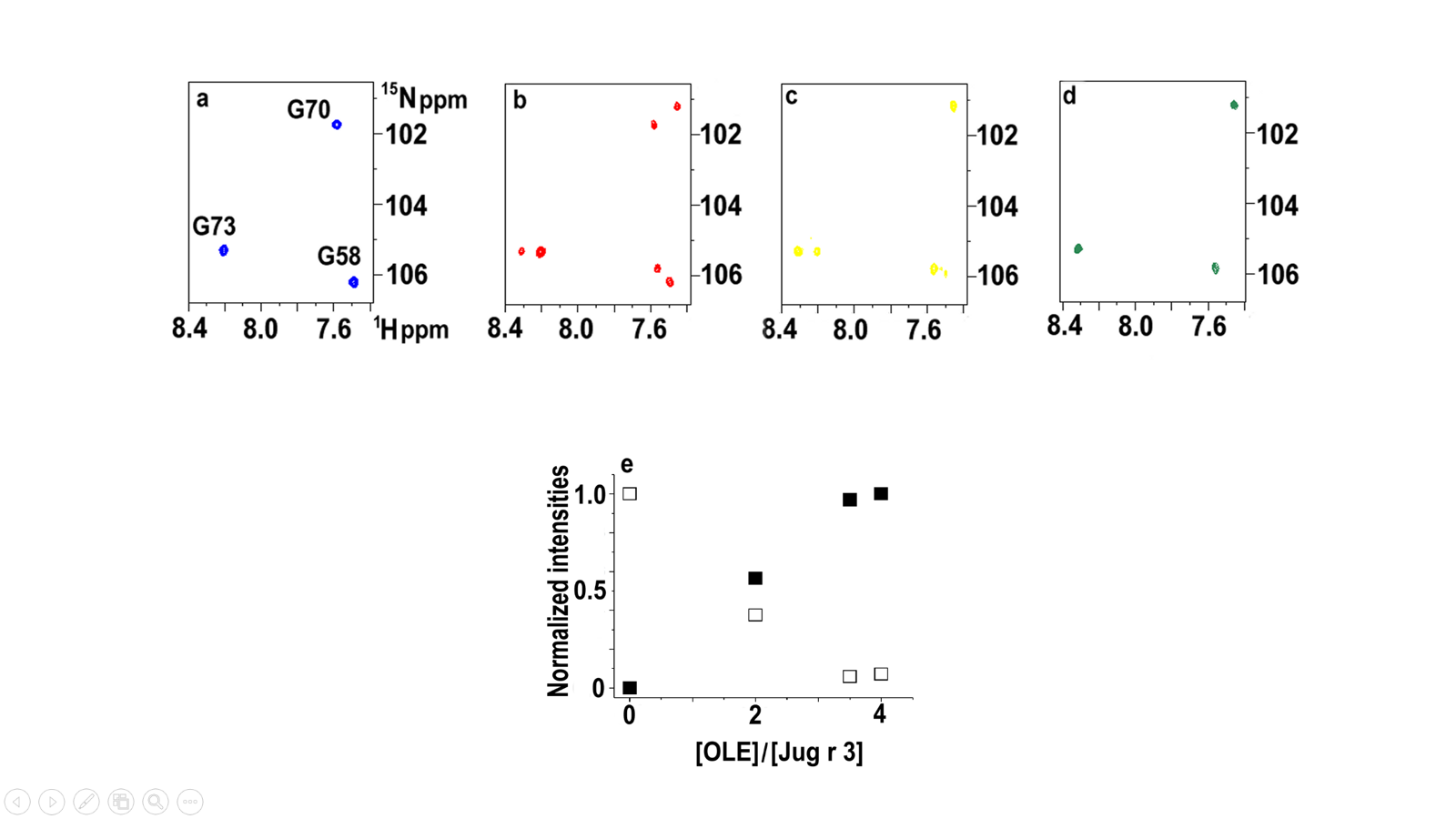


**Supplementary Figure 3:** Titration of Jug r 3 with oleate. A selected region of ^1^H-^15^N HSQC spectra recorded at [OLE]/[Jug r 3] ratio equal to **a**) 0; **b**) 2; **c**) 3.5 and **d**) 4, showing amide resonances in slow exchange on the NMR time scale (G70, G73 and G58). **e**) Resonance intensity ratio of OLE-bound vs. OLE-free Jug r 3 for Gly 70 (I_OLE-bound_/(I_OLE-free_) plotted against the [OLE]/[Jug r 3] ratio.


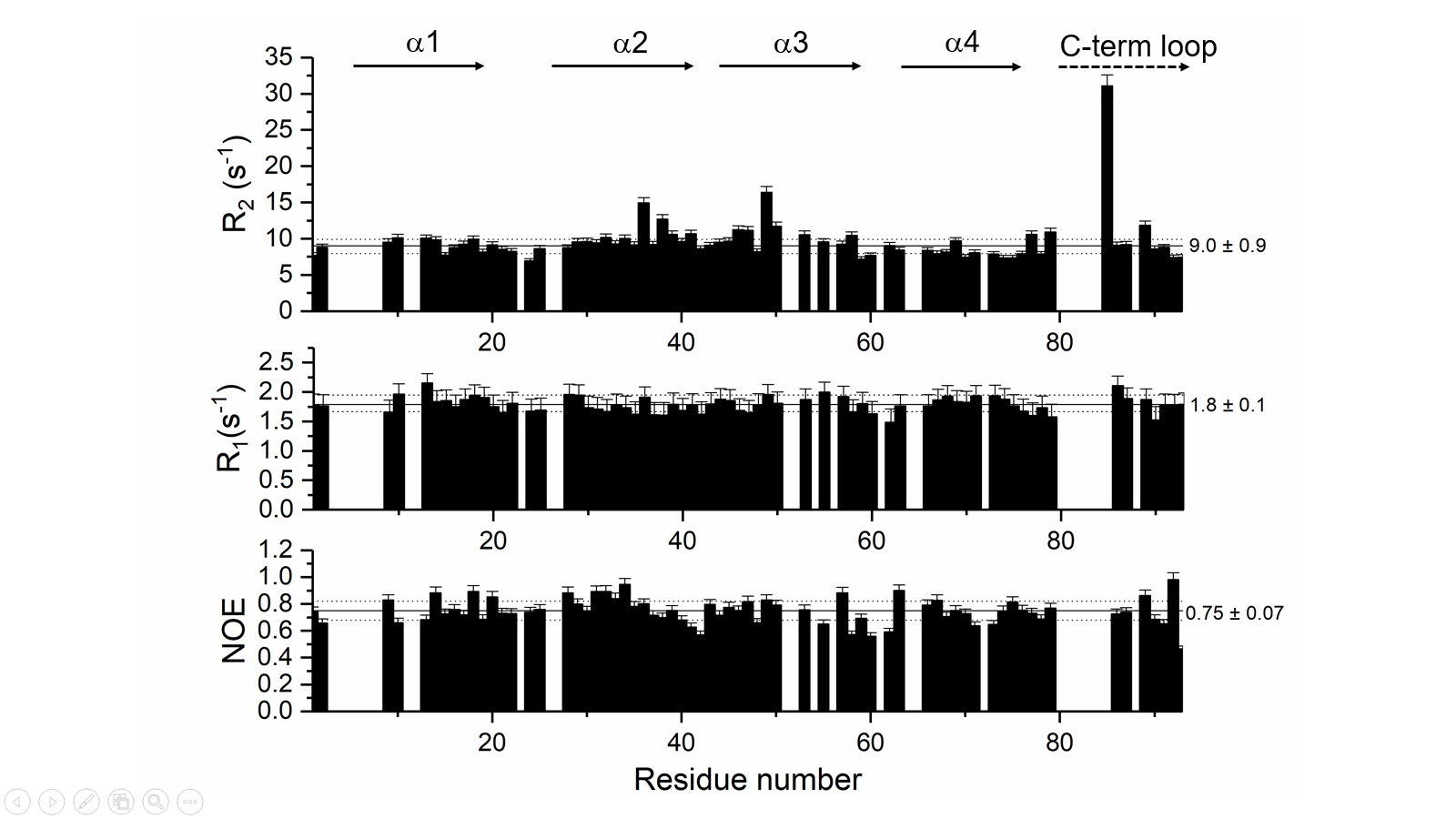


**Supplementary Figure 4:** **Internal mobility of OLE-bound Jug r 3 protein**. ^15^N transverse (R_2_) and longitudinal (R_1_) relaxation rates and ^15^N{^1^H}-NOEs recorded at 298 K at 700 MHz, using a ^13^C,^15^N labelled sample (0.2 mM) are shown. The lines reported in the graphs represent the average values ± one standard deviation. The secondary structure elements are reported at the top. The ^15^N longitudinal relaxation rates (R_1_) were measured with delays of 10, 40, 75, 80, 125, 200, 370, 500, 675, 800, 1000, 1500 and 2500 ms. The ^15^N transverse relaxation rates (R_2_) were measured using a CPMG sequence with delays of 16.96, 33.92, 50.88, 67.84, 101.76, 135.68, 203.52, 237.44, 254.40, 288.32, 339.20, and 373.21 ms and a refocusing time (τ_CPMG_) of 450 μs. In both R_1_, R_2_ measurements, the first delay was duplicated. A recycle delay of 3 s was used for R_1_ and R_2_ and of 5 s for the ^15^N{^1^H}-NOE.
